# An improved and simplified somatic embryogenesis protocol in Chir pine (*Pinus roxburghii)*

**DOI:** 10.1038/s41598-025-20364-4

**Published:** 2025-10-28

**Authors:** Farah Kousar, Ulrika Egertsdotter, Faheem Aftab

**Affiliations:** 1 Institute of Botany, University of the Punjab, Lahore, 54590 Pakistan; 2 Department of Forest Genetics and Plant Physiology, Swedish Agriculture University, 90734 Umea, Sweden

**Keywords:** Conifer, Embryogenic tissue, Maturation, Somatic embryos, Western himalayan mountains, Biotechnology, Molecular biology, Plant sciences

## Abstract

Forests are considered to be an important pillar for a country’s bioeconomy but unfortunately are being depleted globally. *Pinus roxburghii* has considerable ecological and economic importance for Pakistan. It is successfully used as a pioneer species in afforestation and reforestation programs in the era of climate change. This paper reports on a significantly improved protocol for *P. roxburghii* somatic embryogenesis. Successive developmental stages for zygotic as well as somatic embryos were reported first time for this important pine species. Initiation of somatic embryogenesis was affected by the developmental stage of the zygotic embryo. Immature pre-cotyledonary zygotic embryos produced 33% initiation of embryonic masses on LP-889 medium supplemented with 11 µM Naphthaleneacetic acid (NAA), 2.5 µM Benzylaminopurine (BAP), 2.5 µM kinetin and 4 µM Abscisic acid (ABA). LP-1250 medium provided with 2 µM BAP, 2 µM kinetin, 5 µM 2, 4-Dichlorophenoxyacetic acid (2, 4-D) and 5 µM ABA was used for the proliferation and maintenance of embryogenic tissue. Polarized immature somatic embryos were clearly visible during the proliferation and maintenance phase. Two media; mLV and LP-1562 were tested as well as several combinations of L-glutamine and ABA for maturation. Maximum number of somatic embryos (310 embryos per g embryogenic tissue) was obtained on LP-1562 medium (containing 450 mg/l of L-glutamine and 20 µM ABA) as compared to 204 embryos per g embryogenic tissue on mLV medium (containing 900 mg/l L-glutamine and 60 µM ABA). Mature somatic embryos were formed on maturation medium after 6–8 weeks. Successful conversion of somatic embryos resulted after 12 weeks on LP-397 medium. However, low rooting rates (7% and 3%) were observed for somatic embryos formed on maturation media, i.e. LP-1562 and mLV, respectively and precocious germination of the mature somatic embryos still remains a serious constraint.

**Supplementary Information:**

The online version contains supplementary material available at 10.1038/s41598-025-20364-4.

## Introduction

Somatic embryogenesis (SE) is considered a more advanced and promising technique than other vegetative propagation methods used for conifers^1^. A lot of advancements and improvements are being introduced in SE protocols by scientists, though, for many conifer species, SE is still difficult or achieved with low efficiency levels^2^. SE has been reported in pine spp. with different success rates, e.g. in *Pinus taeda*^3^, *Pinus pinaster*^4,5^, *Pinus oocarpa*^6^ and *Pinus strobus*^7^. Studies highlighted that staging zygotic embryo development was regarded as one of the key factors to obtain maximum initiation^8,7,9,10^. Little attention has been paid to improve the SE protocol in *Pinus roxburghii* by focusing on the developmental stage of the zygotic embryos. A very low initiation rate (0.5-1.0%) has been reached by using immature zygotic embryos in *P. roxburghii*^11,12^. A successful attempt was made in 2011 to induce embryogenic cultures from the same explants^13^. Unfortunately, many aspects of this work remain unpublished. A successful SE protocol has been devised for *P. taeda* by Pullman and co-workers^3,14–16^ by using zygotic embryos. Hence, in the present study for aforementioned reasons, we have attempted to extend the work on *Pinus taeda* for the establishment of a workable SE protocol for *Pinus roxburghii*.

Our specific objectives in the present investigation included identification of the most suitable immature precotyledonary stage of zygotic embryos for SE culture initiation and optimization of maturation media to refine in vitro SE success rates in this economically important pine species that is *P. roxburghii*.

## Materials and methods

### Plant material

**Fig. 1:**
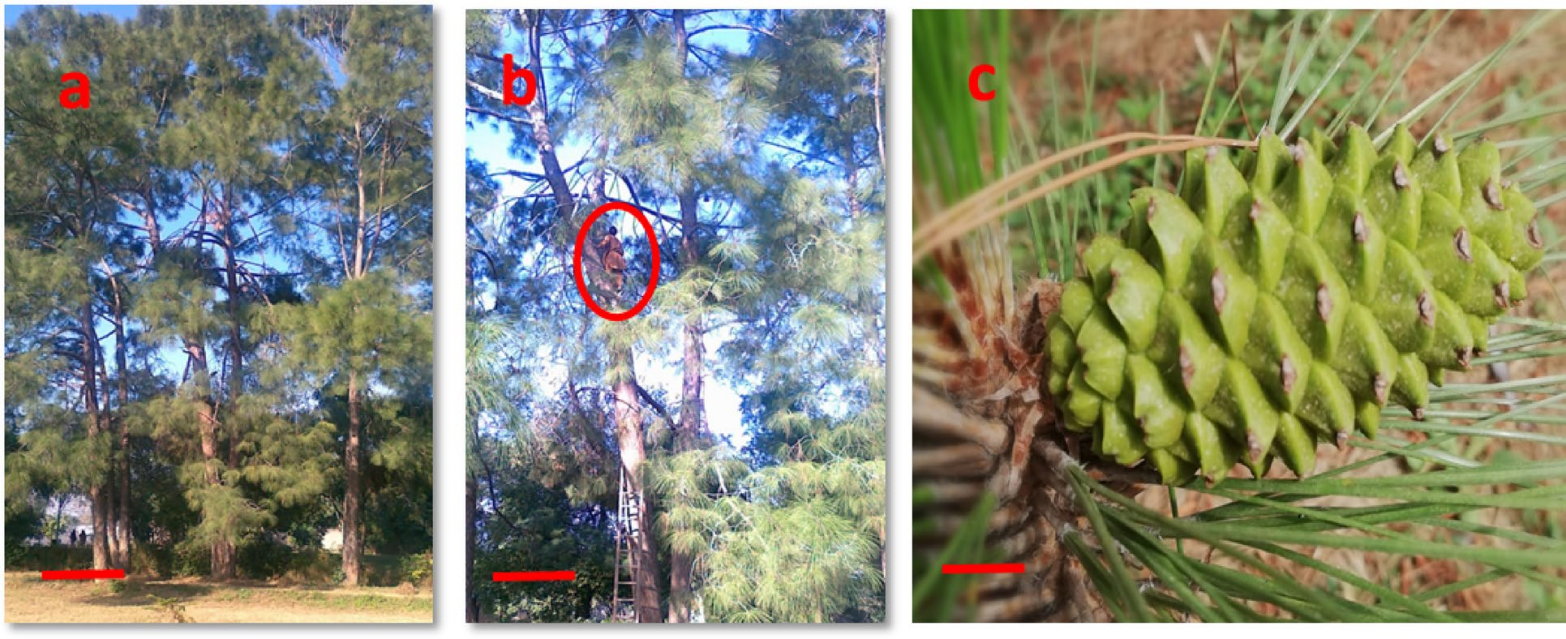
*Pinus roxburghii* selected as mother trees (**a**). A worker climbing on pine tree (encircled) for collection of cones (**b**). Immature female cone of *Pinus roxburghii* collected on 20th June (**c**). (Scale Bar a = 5 feet, b = 4.8 feet, c = 1.5 cm).

Immature cones of *P. roxburghii* of open pollinated trees were collected after every two days from June 20th to July 11th for three years (2016–2018) from Botanical Garden, University of the Punjab, Lahore, Pakistan. The *P. roxburghii* orchard in this location (31ºN, 74ºW) was established in the early 1960s. An ample number of cones were collected from seemingly physiologically healthy three pine trees selected as mother plants for the current study Fig. 1(a-c). The cones were washed and sprayed with 70% ethanol. Extracted seeds were washed with distilled water for 10 min. The seeds were agitated in 10% (v/v) house-hold bleach (containing 5% sodium hypochlorite NaOCl) in combination with 0.2% (v/v) Tween-20 for 10 min and then rinsed with distilled water for 30 min and treated with 20% (v/v) hydrogen peroxide (H_2_O_2_) for 10 min. Finally, seeds were washed with autoclaved distilled water 3–4 times in a laminar air flow hood. The zygotic embryo (ZE) stages were compared to Cairney and Pullman^8^ staging system and numbers were assigned accordingly.

Experimental work, including initiation and proliferation of SE cultures, was performed at the Plant Developmental and Regenerative Biology lab, Lahore, while maturation and conversion of somatic embryos experiments were conducted in SE-Lab, Umeå Plant Science Centre, Umea, Sweden. All experiments were performed following relevant guidelines and regulations.

### Initiation of embryogenic tissue

Five excised megagametophytes (MGs) containing immature zygotic embryos (ZEs) were placed horizontally in each Petri plate (60 mm × 14 mm) containing modified LP (889) medium^14^ provided with 11 µM NAA, 2.5 µM BA, 2.5 µM kinetin and 4 µM ABA and pH of the medium (5.7) was adjusted with 1 N KOH or 1 N HCl after the addition of all the components except gelling agent and filter sterilized growth regulators. Growth regulators and aqueous solution of L-glutamine were filter-sterilized with a 0.22 μm pore size filter membrane and then added to the autoclaved (at 121 °C at 15 lbs inch^− 2^ for 20 min) medium after being cooled to approximately 55 °C. L-Asparagine and 8-Br-cGMP was omitted in modified LP 889 medium^14^.

Each year, on each collection date, 150 MGs (50 MGs from each of three selected trees) were inoculated resulting in a total of 1200 MG inoculations annually. All the inoculated MGs containing immature ZEs were placed in a dark room under controlled conditions of temperature (25 ± 1 °C) for 2–3 weeks.

Early extrusion was scored when the suspensor region of the ZE pushed out at the micropylar region of the megagametophyte. Initiation was considered as a stage when late extrusions showed continuous growth and produced enough embryogenic tissue for subculture and continued growth of the isolated embryogenic tissue. Each isolated initiation was considered a cell line. The percentage initiation was recorded for three consecutive years (2016, 2017 and 2018). Extrusions and initiations were observed on the same medium without any subculture. These were recorded during 2–3 weeks after inoculation on the medium.

### Proliferation of the embryogenic masses

After initiation, proliferating embryogenic masses were separated from megagametophytes and transferred to modified LP 1250 maintenance medium^3^ supplemented with 2.0 µM BAP, 2.0 µM Kinetin, 5.0 µM 2, 4-D and 5 µM ABA (MES, Biotin and folic acid were not included in modified LP-1250 medium) for multiplication of the early-stage somatic embryos in the embryogenic tissue. Embryogenic tissue (200 mg) from each megagametophyte was considered as initiation and subcultured on proliferation medium contained in petri plates (90 mm × 15 mm). Successful initiations had a good multiplication potential. The cultures were placed in the dark room under controlled conditions (25 ± 1 °C). Sub-culturing was done fortnightly onto fresh maintenance medium. The increase in fresh weight (FW) of the embryogenic tissue was recorded after every 4 days for each cell line (CL) for 20 days. To measure the increase in fresh weight, cell lines were weighed aseptically in sterile petri plates under a laminar air flow hood by using a digital weighing balance. Clumps were weighed aseptically and thereafter placed back to the corresponding medium. Ten replicates for each CL in a separate petri plate (60 mm × 14 mm) were prepared. Proliferating cell lines (CL-1, CL-2 and CL-3) included in the further experiments were taken from the year 2018 initiations.

### Maturation of somatic embryos

Maturation experiments were performed on the LP-1562 medium in Pakistan during 2016 and 2017. For optimization, CLs were raised in July 2018 in Pakistan and brought to Sweden for use in further experiments performed at SE Lab Sweden during May 2019. Fresh two subcultures were done prior to maturation experiments. Results obtained on LP-1562 medium in the past (2016 and 2017) were in line with the repeated experiments with LP-1562 medium (yield presented in results section) in SE LAB, Sweden. Therefore, LP medium was used as a control medium and different combinations of ABA and L-glutamine were used for the mLV medium^17^ which was successfully used for somatic embryogenesis of *Pinus pinaster*^18^, moreover both pine species (*P. roxburghii* and *P. pinaster*) are closely related to each other and share many features^19^. Three embryogenic cell lines (CL-1, CL-2 and CL-3) derived from the three different trees were selected based on their in vitro multiplication potential, very low to negligible necrosis rates and their capacity to form mature somatic embryos. Pre maturation treatment was given to somatic embryos prior to maturation step. For pre maturation treatment, three (200–300 mg) clumps of proliferating embryogenic tissue were transferred to each Petri plates (60 mm × 14 mm) of pre-maturation medium composed of maturation medium LP 1562^3^ or mLV^17^ each without ABA. After 10 days, the clumps of embryogenic masses were transferred to maturation medium with different combinations of ABA and L-glutamine (Fig. 9 ). Three replicates of each cell line were prepared for each treatment. Plates were sealed with parafilm^®^ M (Bemis Company, Inc.) and incubated at 23 °C in the dark for 6–8 weeks. The development of somatic embryos was observed with a stereomicroscope (Wolfe^®^ microscopes, USA).

The maturation medium mLV was supplemented with three concentrations of ABA (30, 60 and 80 µM) based on the literature on *Pinus pinaster* whereas the concentrations of L-glutamine tested were based on the concentrations used for *Picea abies*, i.e., 450 mg/L^20^ and 1460 mg/L for *Larix species* (*Larix × eurolepis*, and *Larix × marschlinsii)*^21^ and 625 mg/L for *Pinus pinaster*^18^.

### Conversion of mature somatic embryos

Mature somatic embryos were transferred to the LP-397^3^ and MS^22^ medium supplemented with 10 µM IBA^23^ in 150 × 20 mm Petri plates under red light at 5 µmol m^˗2^ s^˗1^ (Philips TLD Red 18 W). Root number per plantlet, root and shoot length were recorded for three months.

### Statistical analysis

Data were analyzed using one-way and two-way analysis of variance (ANOVA) followed by Duncan’s multiple range test (DMRT) to find out the mean difference and homogeneity amongst the tested variables at *p* < 0.05% significance level by using SPSS v 20. Prior to ANOVA Levene’s test was also performed to assess the equality of variances.

## Results

### Development of zygotic embryos

**Fig. 2:**
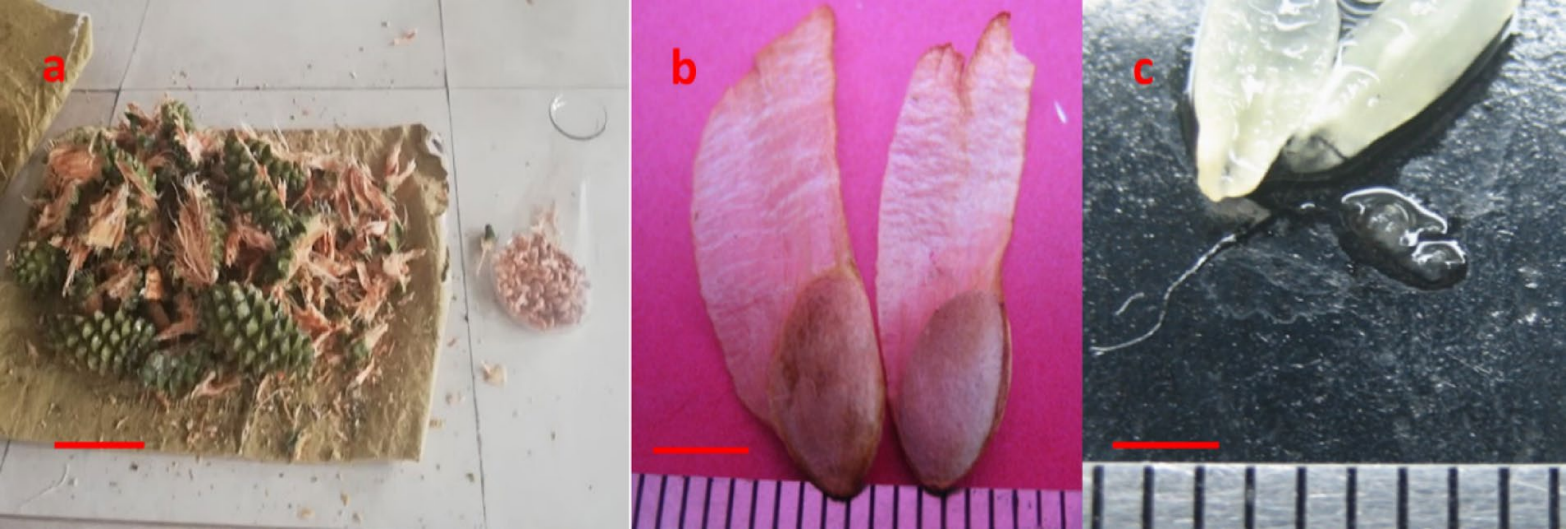
Extraction of seeds from *P. roxburghii* cones (**a**). Mature seeds with papery wing extracted from the cones collected on 30th September (**b**). Primary suspensor tissue from the megagametophyte collected on 18th June (**c**). (Scale Bar a = 5 cm, b = 5 mm, c = 2 mm).

**Fig. 3:**
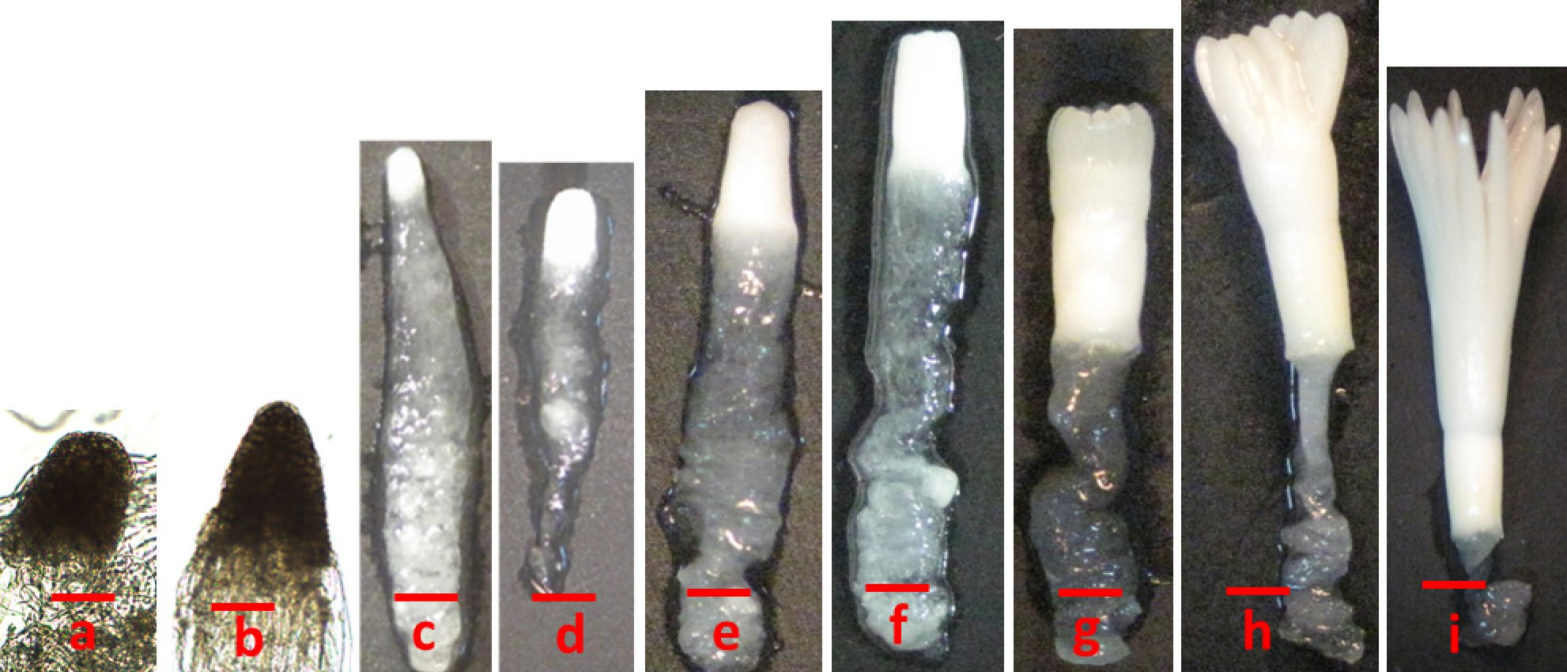
Maturation of zygotic embryos in *Pinus roxburghii.* Polyembryony was observed in the 23rd June collection (**a**). On 29th June collection, early dominance of one embryo was seen (**b**). 2nd July cone collection shows precotyledonary stages of ZEs (**c-e**) with prominent apical dome shaped heads. Early cotyledonary stages of ZEs were observed on 7th July (**f**,** g**). Cotyledons completely covered the apical primordium of ZEs in the cones collected on 20th July (**h**,** i**). (a, b Bar = 200 μm, c-i Bar = 500 μm). Numbering convention based upon Cairney and Pullman^8^.

The developmental stages of zygotic embryos were different for each collection date ranging from proembryo stage to the cleavage polyembryony and formation of the dominant cotyledonary embryo stages Fig. 2(a-c) and Fig. 3(a-i). Cones collected on 18th June contained zygotic embryos with a primary suspensor Fig. 2(c) while polyembryogeny was observed in the cone collection of 23rd June Fig. 3(a). On 29th June, post-cleavage stage with the early dominance of one embryo was seen Fig. 3(b). Dominance of one zygotic embryo head started in the first week of July Fig. 3(c). Embryo stages shown in Fig. 3(c-e) were considered as pre-cotyledonary stages having relatively longer suspensor regions with prominent apical dome shaped heads. It was noted that the ZEs present in the single female cone were not at the same developmental stage as can be predicted based on the polyembryonic process. Early cotyledonary embryo stages shown in Fig. 3(f, g) were first observed on 7th of July and apical primordium was visible in some ZEs or partially covered by cotyledons. Embryo stages shown in Fig. 3(h, i) were present in cones collected on 20th July. Cotyledons completely covered the apical primordium of ZE at these stages Fig. 3(i).

### Extrusion and initiation

**Fig. 4:**
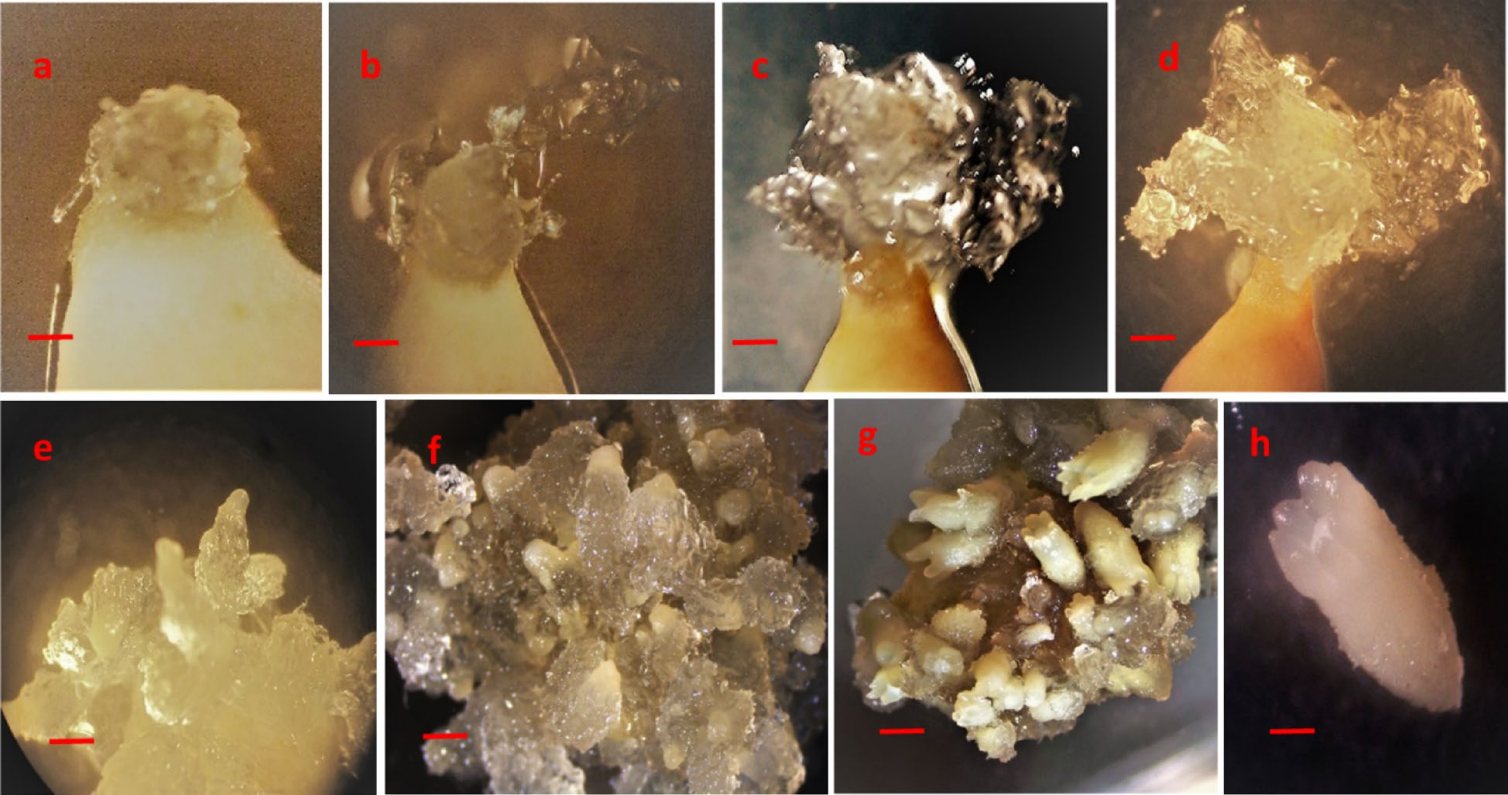
Stages of somatic embryogenesis in *P. roxburghii.* Extrusions from the megagametophyte were observed during the first week of inoculation on the initiation medium (**a-c**). During 2–3 weeks of inoculation, initiation of ET was observed (**d**), proliferation of ET was observed to be in the form of clumps of immature somatic embryos and achieved by subculturing fortnightly (**e**), early maturation treatment was given for 10 days (**f**), and mature somatic embryos were formed on maturation medium in 6–8 weeks (**g**,** h**) (Bar = 500 μm).

**Fig. 5:**
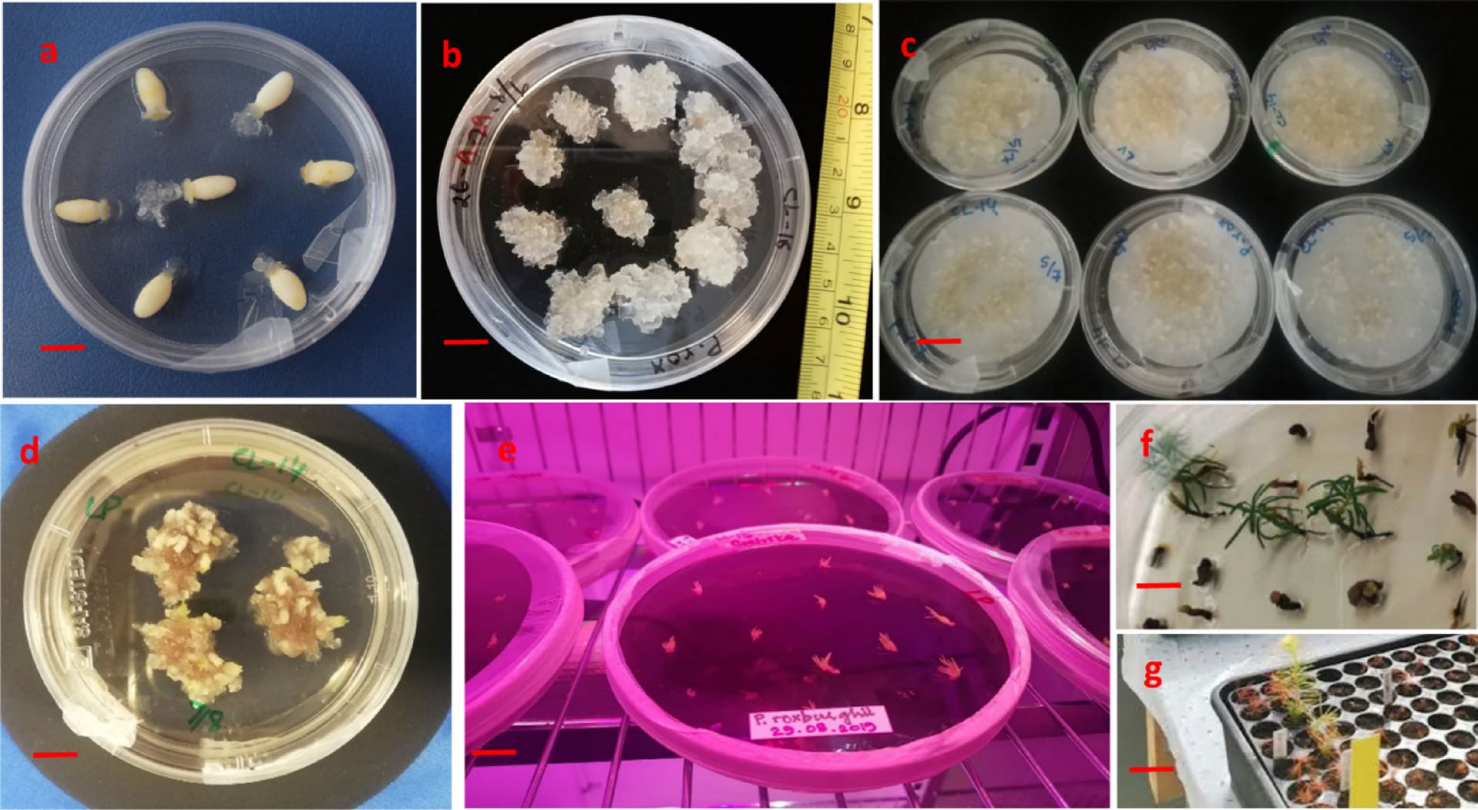
Sequence of developmental steps involved in somatic embryogenesis of *P. roxburghii*. Extrusion and initiation of ET (60 mm × 14 mm petri plate) were observed for 2–3 weeks (**a**), proliferation (90 mm × 15 mm petri plate) was achieved by subculturing on fresh medium fortnightly (**b**), maturation step takes 6–8 weeks (**c**,** d**) and conversion of somatic embryos observed for three months in 150 × 20 mm petri plates (**e**), germinated somatic embryo (**f**,** g**). (Scale: a-c Bar = 1 cm, d Bar = 0.8 cm, e Bar = 1 cm, f Bar = 2 mm, g Bar = 2 cm).

**Fig. 6:**
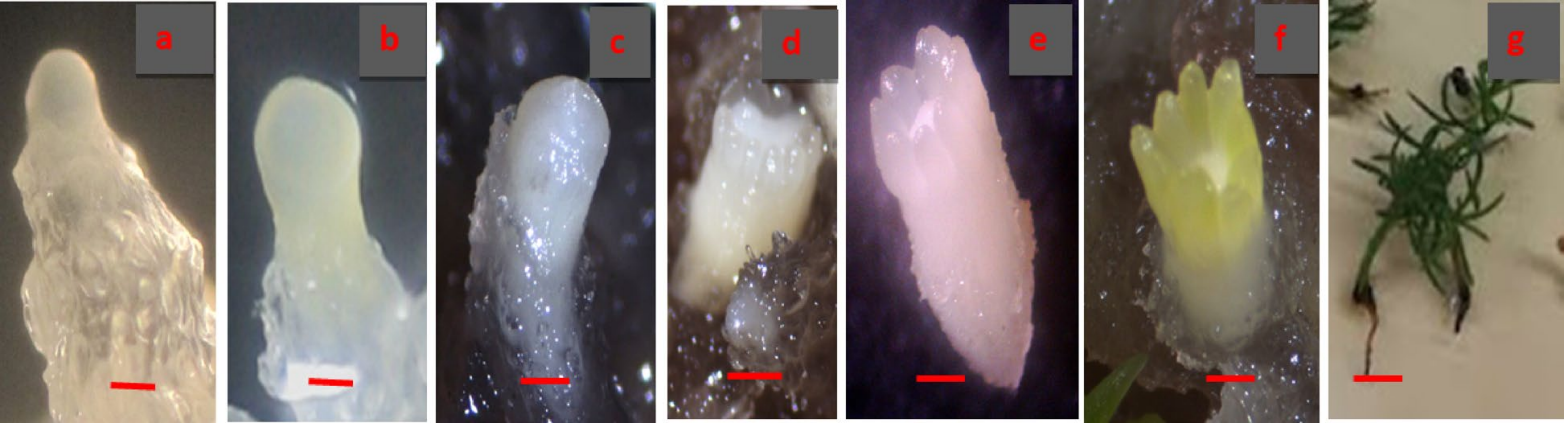
Sequence of developmental stages of somatic embryos during maturation of *Pinus roxburghii* (a-f Bar = 500 μm, g Bar = 2 mm).

Extruded tissue continued its growth Fig. 4(a, b) and formed a proliferating culture of extruded tissue defined as Extrusion Fig. 4(c). Extrusions from all attempted initiation events were observed during the first week of inoculation. Extruded masses continued their growth in all directions and termed as Initiation Fig. 4(d) and Fig. 5(a). Initiated embryonic masses were spiky, white and translucent in morphology Fig. 4(e). Multiplication of the embryogenic tissue was in the form of clumps of early-stage somatic embryos with opaque heads relative to the suspensor regions that were more translucent and easily distinguishable Fig. 4(e) and Fig. 5(b). Embryos mature in 6–8 weeks on maturation medium Fig. 4(f-h) and Fig. 5(c, d) and three months on conversion of somatic embryos medium Fig. 5(e-g). Different developmental stages of somatic embryos of *Pinus roxburghii* during maturation were observed Fig. 6(a-g).

**Fig. 7:**
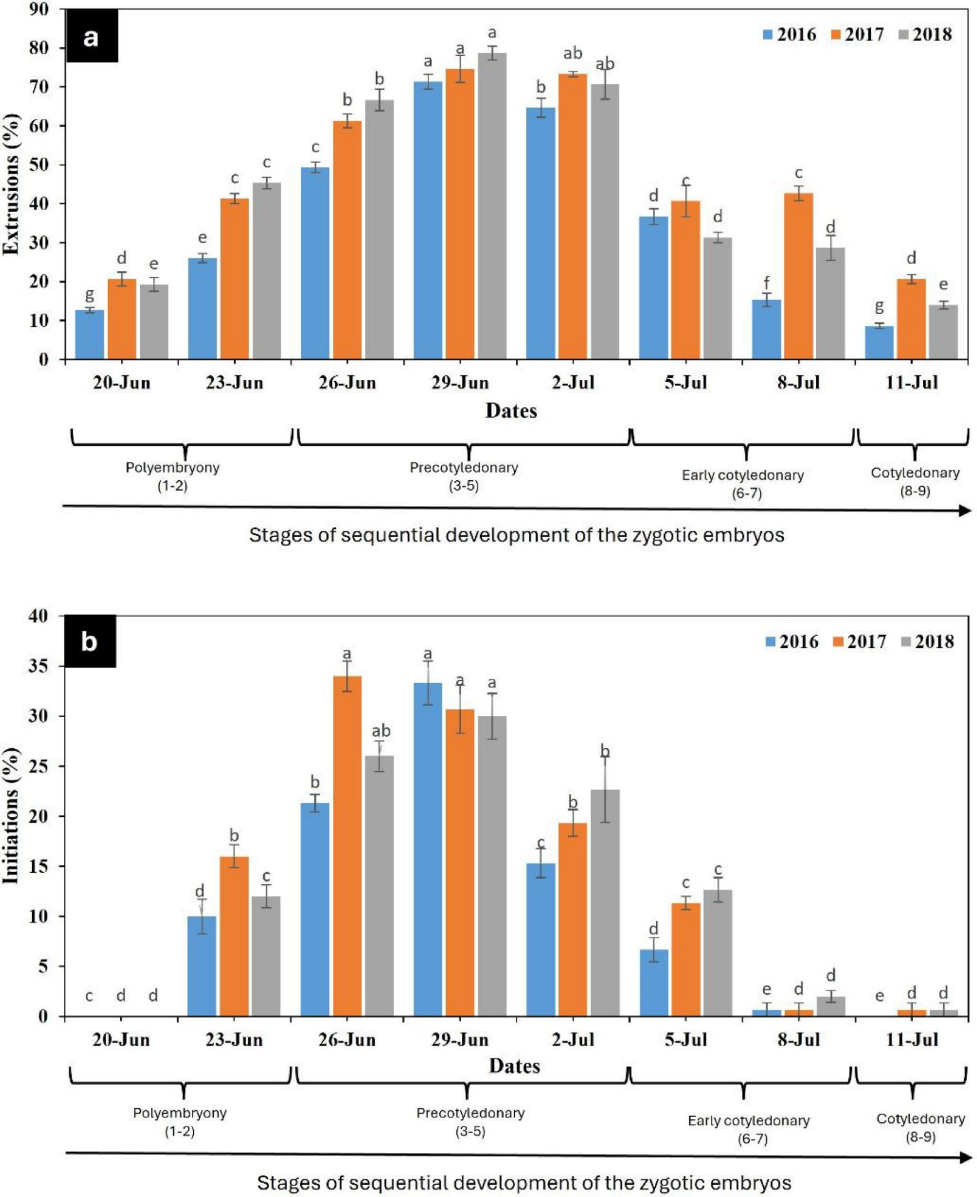
Effect of collection dates on percent values of extrusions (**a**) and initiations (**b**) of embryogenic tissue from developing ZEs of *Pinus roxburghii* during 2016–2018. Each peak represents the mean (%) ZEs response for extrusion and initiation during different collection dates of three year’s experiments. Values (± SE) are means of 30 replications per collection date (5–6 seeds per replicate). Each year, mean percentage extrusions and initiations at different collection dates were compared based on Duncan’s multiple range test and significant differences at *p* < 0.05% were indicated by different letters.

During the three consecutive years, there was a significant correlation between mother trees and collection dates, but extrusion and initiation rates showed significant differences when collection dates were taken into account, Fig. 7(a, b). Early response for extrusions started from 20th June and was at its maximum on 29th June collection date, Fig. 7(a), while early response for initiations was noted from 23rd June, Fig. 7(b). The highest number of extrusions (78.7%) and initiations (33.34%) were recorded on 29th June. Fewer extrusions and a very low number of initiations were obtained after 5th July, Fig. 7(a, b). According to findings, for three consecutive years, there were overall mean extrusions of 35.6%, 46.9%, and 44.3%, and initiations of 10.92%, 14.08%, and 13.25%, respectively.

### Proliferation

**Fig. 8:**
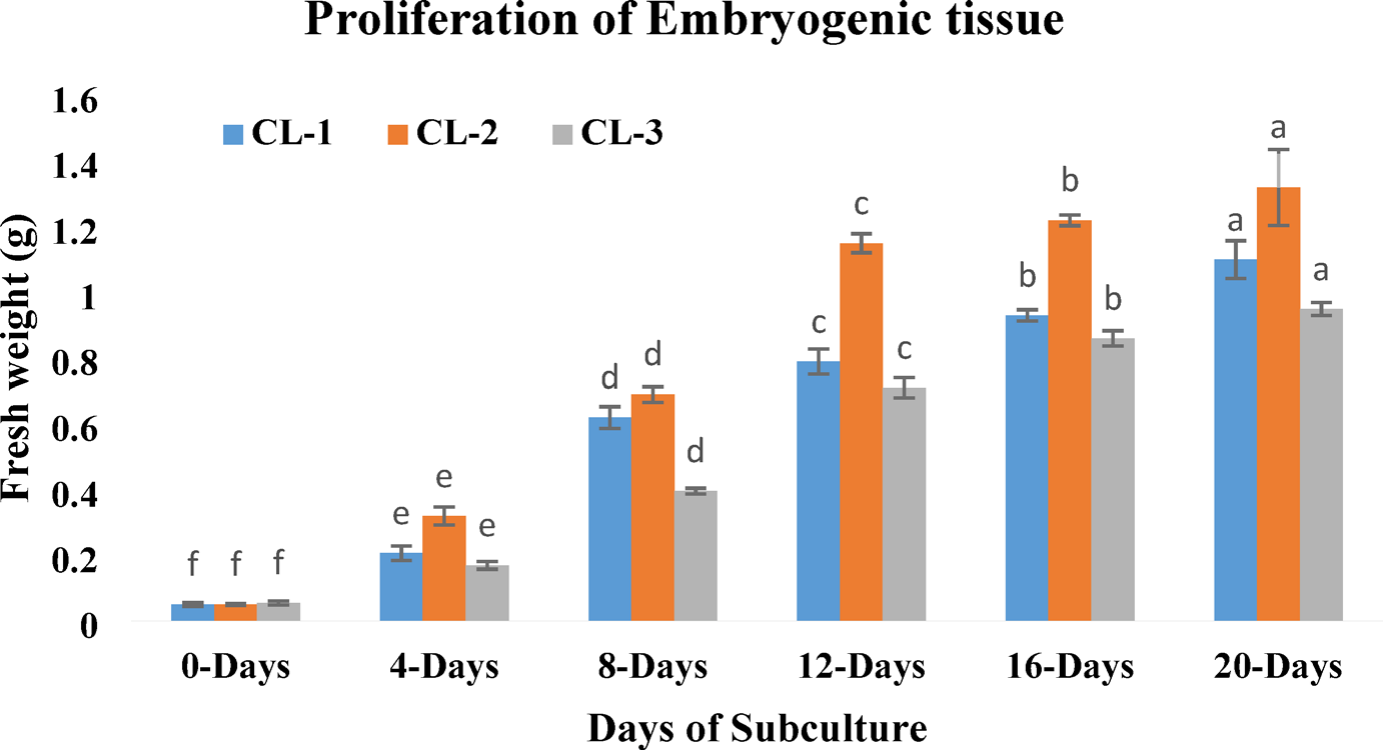
Increase in fresh weight of embryogenic tissue (starting from 0.05 g per cell line) of *P. roxburghii* over the period of 20 days during the proliferation phase. Data represent mean (± SE) from three replicates for each cell line (CL) (each replicate contain 5 clumps). Fresh weight increase for each cell line was compared based on Duncan’s multiple range test and significant differences at *p* < 0.05% were indicated by different letters.

An increase in the fresh weight of embryogenic tissue was recorded for the three cell lines (CL-1, CL-2 and CL-3). During the first 12 days, the growth rate was very high (Fig. 8); however, the multiplication rate was not that high after two weeks of subculture. A higher rate of proliferation was observed in the CL-2 as compared to the other cell lines (CL-1 and CL-3).

### Maturation

**Fig. 9:**
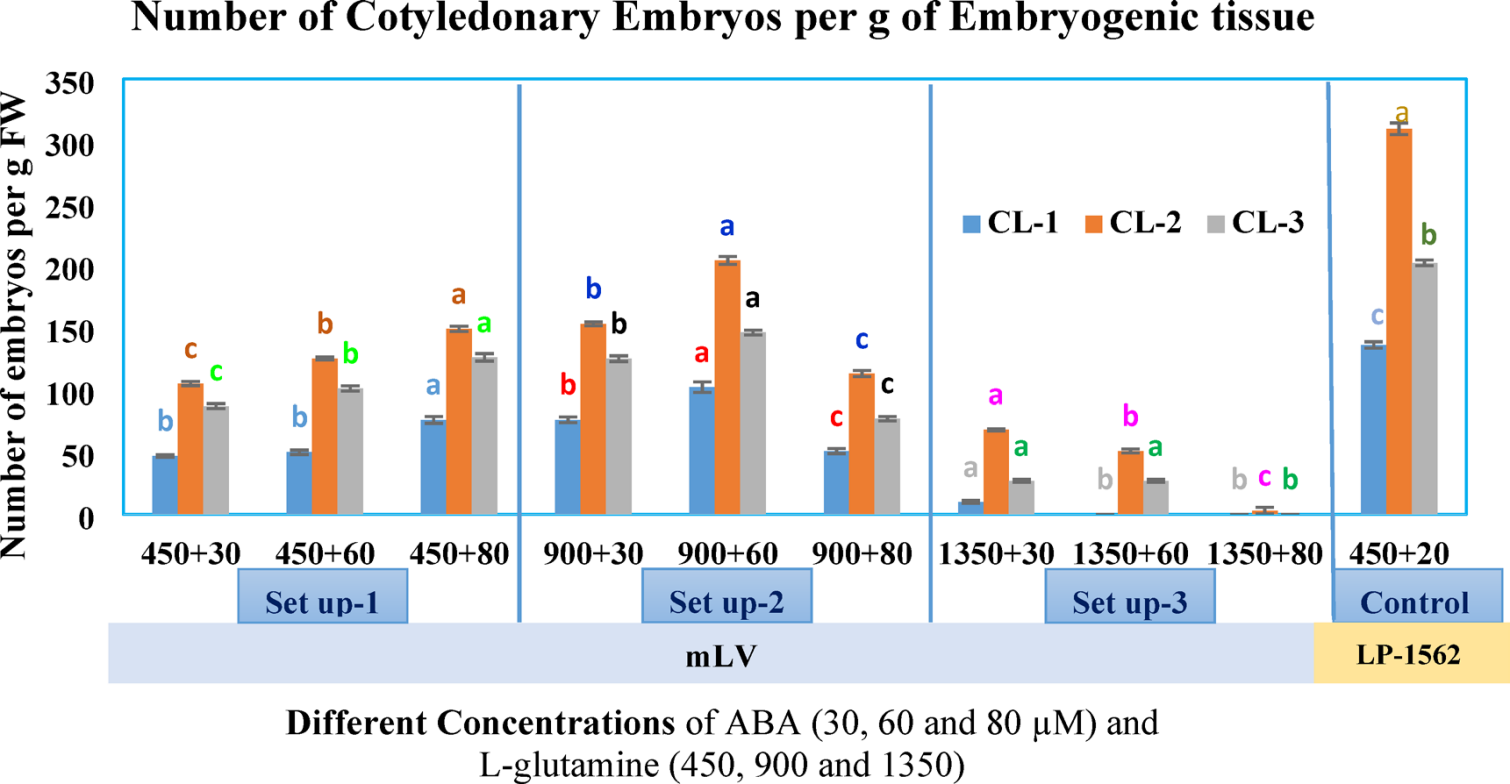
Effect of different concentrations of L-glutamine, ABA and of two media (mLV and LP-1562) on the number of embryos formed per g embryogenic tissue of *P. roxburghii*. Each peak represents the mean number of somatic embryos. Values (± SE) are means of 15 replications (five clumps per replicate). Different concentrations of L-glutamine were tested based on the concentrations used for Norway spruce (450 mg/L^20^, *Larix species; Larix × eurolepis*, and *Larix × marschlinsii)* (1460 mg/L^21^ and *Pinus pinaster* (625 mg/L^18^. The concentrations of ABA were designed keeping in view the literature on *Pinus pinaster*^16,18,24^. In each experimental set-up for each cell line different treatment means were compared based on Duncan’s multiple range test and significant differences at *p* < 0.05% were indicated by different letters.

Embryos were formed on maturation medium after 6–8 weeks. Embryo yield showed a significant difference when different concentrations of L-glutamine and ABA were considered (Table 1). LP-1562 supported higher embryo yield than the medium mLV (Fig. 9). CL-2 produced more embryos (310 embryos per g ET) as compared to other CLs on mLV basal medium with 900 mg/l L-glutamine and 60 µM ABA. A maximum number of embryos was also produced by CL-2 on mLP-1562 medium (204 embryos per g ET) as compared to other CLs. A few or no mature somatic embryos were obtained in any of the three cell lines when higher concentrations of L-glutamine and ABA were used.

**Table 1:** Two-way ANOVA (multivariate analysis) for interactive effects of Glutamine and ABA on three selected Cell Lines of *P*. roxburghii during maturation.

Source	Dependent Variable	Df	F value	Sig. <0.05	
Corrected Model	CL1	8	13.713	0.000	
	CL2	8	23.873	0.000	
	CL3	8	17.107	0.000	
Intercept	CL1	1	195.213	0.000	
	CL2	1	675.156	0.000	
	CL3	1	350.000	0.000	
Glutamine	CL1	2	44.373	0.000	
	CL2	2	71.418	0.000	
	CL3	2	58.738	0.000	
ABA	CL1	2	0.973	0.397	
	CL2	2	7.631	0.004	
	CL3	2	2.167	0.144	
Glutamine × ABA	CL1	4	4.753	0.009	
	CL2	4	8.221	0.001	
	CL3	4	3.762	0.022	

### Conversion of somatic embryos

**Fig. 10:**
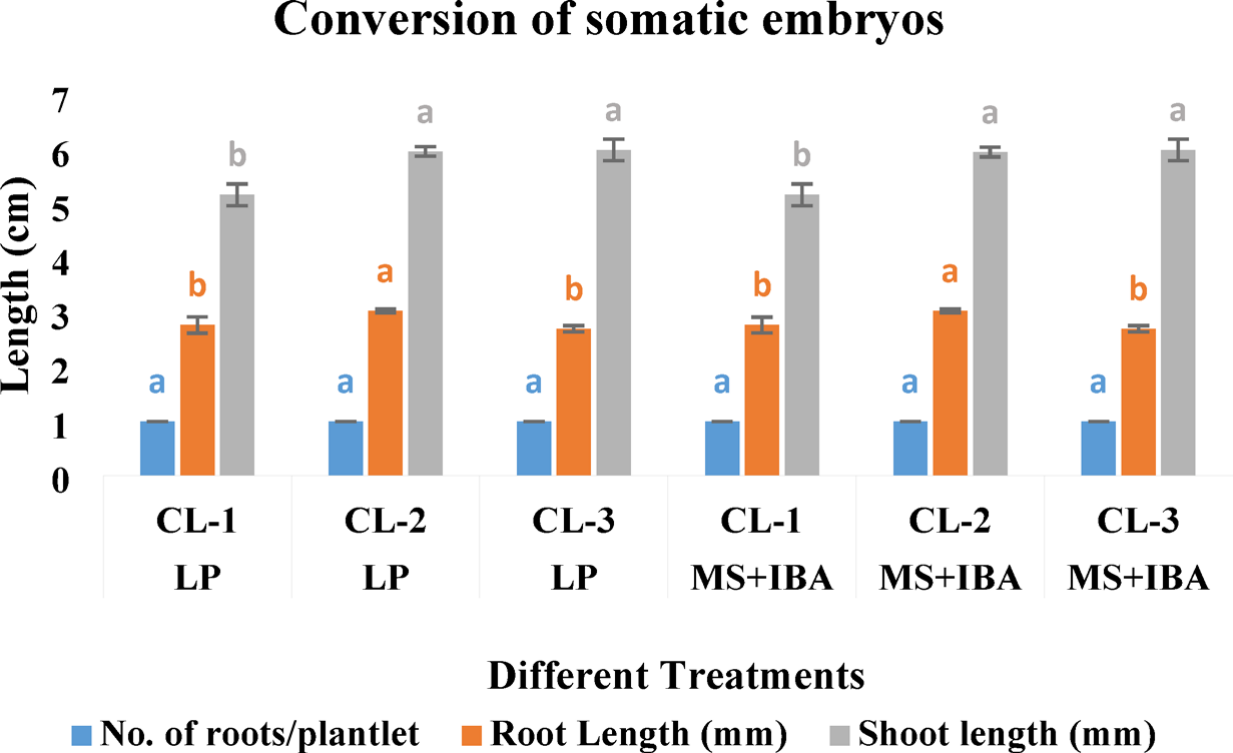
Effect of culture media LP-397^3^ and MS^22^ on the conversion of somatic embryos of *P. roxburghii*. Each peak represents the mean conversion parameter. Values (± SE) are means of 03 replications (10 embryos per replicate). Mean conversion parameters of three cell lines at different treatments were compared based on Duncan’s multiple range test, and significant differences at *p* < 0.05% were indicated by different letters.

Embryos obtained from the maturation experiment were treated for their conversion to form plantlets (Fig. 5e-g). Shoot growth occurred in all embryos, and needles were formed. An almost similar growth pattern was observed for the three selected CLs in both media (Fig. 10). In this study, less than 10% of all the somatic embryos formed on both maturation media developed roots. Some 7% of somatic embryos formed roots on LP-1562 medium as compared to LV medium, which favoured 3% rooting. However, further development of roots was not very encouraging to sustain the plantlets thus developed.

## Discussion

Several reports on somatic embryogenesis in *Pinus roxburghii* have already been presented during the first decade of the 21st century. Different methods used were unfortunately proven not to be reproducible for the same or the other pine species^25^. The lack of reproducible methods may have contributed to the subsequent ten years of void in new research results on SE in *P. roxburghii.* Several pine species have been explored by different research groups, for example *Pinus pinaster*^4^, *Pinus pinea*^9^ and *P. taeda*^14^ all showing promising SE potential. Keeping in view *Pinus roxburghii*’s economic importance and its positive SE results in our Laboratory, *Pinus roxburghii* should probably be regarded as a model pine species of the Asia like its American counterpart, i.e., *P. taeda*^3,14,26^ to direct efforts towards development of an efficient SE protocol for this important pine species. Some other pine species of Asian region include *Pinus thunbergii*,* Pinus densiflora*,* Pinus armandii* var. *amamiana*, and *Pinus luchuensis*^27^ which showed an overall average initiation frequency of 2.3%^28–31^. The SE process of *P. roxburghii* reported here also appears more promising due to the response to initiation (by using immature zygotic embryo as a starting plant material), almost negligible loss of embryogenic cultures especially after initiation and good yields of embryo formation, although bottlenecks remain suggesting this pine species could become a model species for pine SE.

It is a well-established fact that SE is affected by many factors including selection of initial (ex)plant material, genotype of the parent tree and media components^7,32,33^. Amongst these, selection of source material for the initiation of SE is one of the key factors for success of SE^8,34^. It was observed in our studies that three different developmental stages of ZEs were present even within a single cone at one time. We also attempted to identify different developmental stages of ZEs of *P. roxburghii* by comparing them to an already reported scaling system for *P. taeda*^35^. Almost identical pattern of ZE development in *P. roxburghii* was witnessed though variation in time frame was also observed compared with *P. taeda*.

The results suggest the profound effect of the initial plant material (stage of zygotic embryo) on extrusion and initiation frequencies of embryogenic tissue. Stage-specific response of the explant for embryogenic tissue can also be observed in other pine species^7,9,36,37^. In our experiments, MGs containing immature ZEs were collected from open-pollinated trees, therefore the exact genetic background of zygotic embryos was not known. The effect of genetic factors cannot be ruled out, even though modifications of other aspects such as identification of zygotic embryo developmental stage and basal medium formulation have resulted in maximum overall mean extrusions (35.6%, 46.9% and 44.3%), and initiations (10.92%, 14.08% and 13.25% respectively) for the three consecutive years.

Not all extrusions were converted into successful initiations. It is interesting to note that the time frame for SE response in *P. roxburghii* was found to be almost the same as reported earlier for *P. pinaster* as well as *P. pinea*^9,37^ because in both species, maximum SE initiation frequencies were observed by the end of June until the first week of July. Our average initiation frequencies (10.92%, 14.08% and 13.25%) were generally in agreement with the previously reported initiation frequency for pine species including *Pinus armandii* (0–20%^30^, *P. brutia* (11.6%^38^ and *Pinus nigra* (10.4%^39^.

According to our findings, the SE response for *P. roxburghii* only lasted for a few days under our local climatic conditions and started in the last week of June which is a period of rapid differentiation from precotyledonary to early cotyledonary phase until the first week of July. Initiation of SE for most *Pinus* species was confined to immature ZEs (pre-cleavage and cleavage embryo stages) because during this narrow window of opportunity consisting of merely a few days ZEs proved to be highly competent for SE^9,40–43^.

Some biochemical, physiological and developmental mechanisms are thought to be responsible for bringing about successful conversion from extrusion to initiation but are still quite unknown, despite many attempts to identify these factors^14,40,44–46^. Generally, the correlation between extrusions and initiations has not been reported but in *P. taeda* higher percentage of extrusions resulted in higher percentage values of initiations^14,47^. It was also observed that percentages of initiation increased when the percentages of extrusion were more than 50%^15^. Differences between frequencies of extrusion and initiation were seen in other pine species i.e., *Pinus monticola* Dougl^48^. In some species, most of the extrusions from the MG could be established as embryogenic lines^37,42^. Occasionally, all the extrusions successfully transformed into initiations, e.g., in *P. armandii*^30^.

In our study, not a single initiation was obtained after the first week of July, which may be related to the fact that the cotyledonary embryos were not suitable explants for embryogenic tissue induction. Initiations are restricted to immature embryos also in *P. taeda*^36^, *P. strobus*^49^, *P. bungeana*^50^ and *P. kesiya*^51^. Interestingly, in this study, the response for early extrusions could be observed in just a few hours (4–5 h) after inoculation provided the megagametophytes were inoculated on the medium on the same collection day. Moreover, once the initiations were established, loss of embryogenic potential or sudden necrosis under maintenance conditions was almost negligible and also the rate of embryogenic tissue multiplication was quite encouraging (subcultured after every 10–12 days to fresh medium). However, the survival of embryonic tissue may partly be dependent on the circumstances related to tissue culture techniques (fluctuations in physical conditions of culture room, in vitro medium conditions, or methods of explant sterilization).

Several factors have been reported to affect somatic embryo maturation, such as genotype, osmotic potential, media components and PGRs^52–54^. Conversion of embryogenic masses to mature embryos was reported to be a very critical phase for the successful completion of maturation^55^. During our maturation experiments, two media were tested for embryo yield. LP-1562 medium proved better than mLV medium. SE maturation was preceded by increasing ABA concentration (21 µM for LP-1562 and 80 µM for mLV) as also reported for *Picea abies*^56^, *Pinus sylvestris*^57^ and *Pinus taeda*^3^. It was observed that the nitrogen, ammonium and calcium content of mLV medium far exceeded that of LP-1562 medium. However, mLV medium did not have a higher somatic embryo yield and quality compared to LP-1562. Our findings are consistent with those of Anil and Rao^58^. However, the conversion phase of somatic embryos remained (and still remains) a serious bottleneck for pines in general (and in this study), as already highlighted in a book chapter by Pullman and Bucalo^3^.

The beneficial effect of L-glutamine as an organic elicitor for somatic embryogenesis has been reported in several studies^59–61^. The current study showed that L-glutamine (900 mg/l) in combination with ABA (60 µM) has promoted the yield of cotyledonary somatic embryos as compared to the other concentrations of glutamine tested during the maturation phase. Further increase in L-glutamine from 900 mg/l to 1350 mg/l resulted in decreased yield of cotyledonary somatic embryos. However, improved yield of mature embryos was obtained in other conifer species, with 1350 mg/l and 2000 mg/l L-glutamine in hybrid larches^21^ and *Abies fraseri*^62^ respectively. Furthermore, LP-1562 medium containing 450 mg/l of L-glutamine and 20 µM ABA was more effective in promoting the development of *P. roxburghii*’s mature embryos than on mLV medium. Similar results for mature embryos on LP-1562 medium (450 mg/l L-glutamine) were also observed in *P. taeda*^3^.

Based on our findings, it appears that the concentration of L-glutamine in the maturation medium has a pronounced effect on embryo yield for this pine species. The yield of mature cotyledonary embryos decreased at 1350 mg/l L-glutamine for all the tested cell lines even with 30 µM ABA. For different pine species, ABA concentrations ranging from 20 µM in *P. taeda*^14^ up to 120 µM in *P. pinaster*^37^ have been reported for successful maturation treatments.

**Fig. 11:**
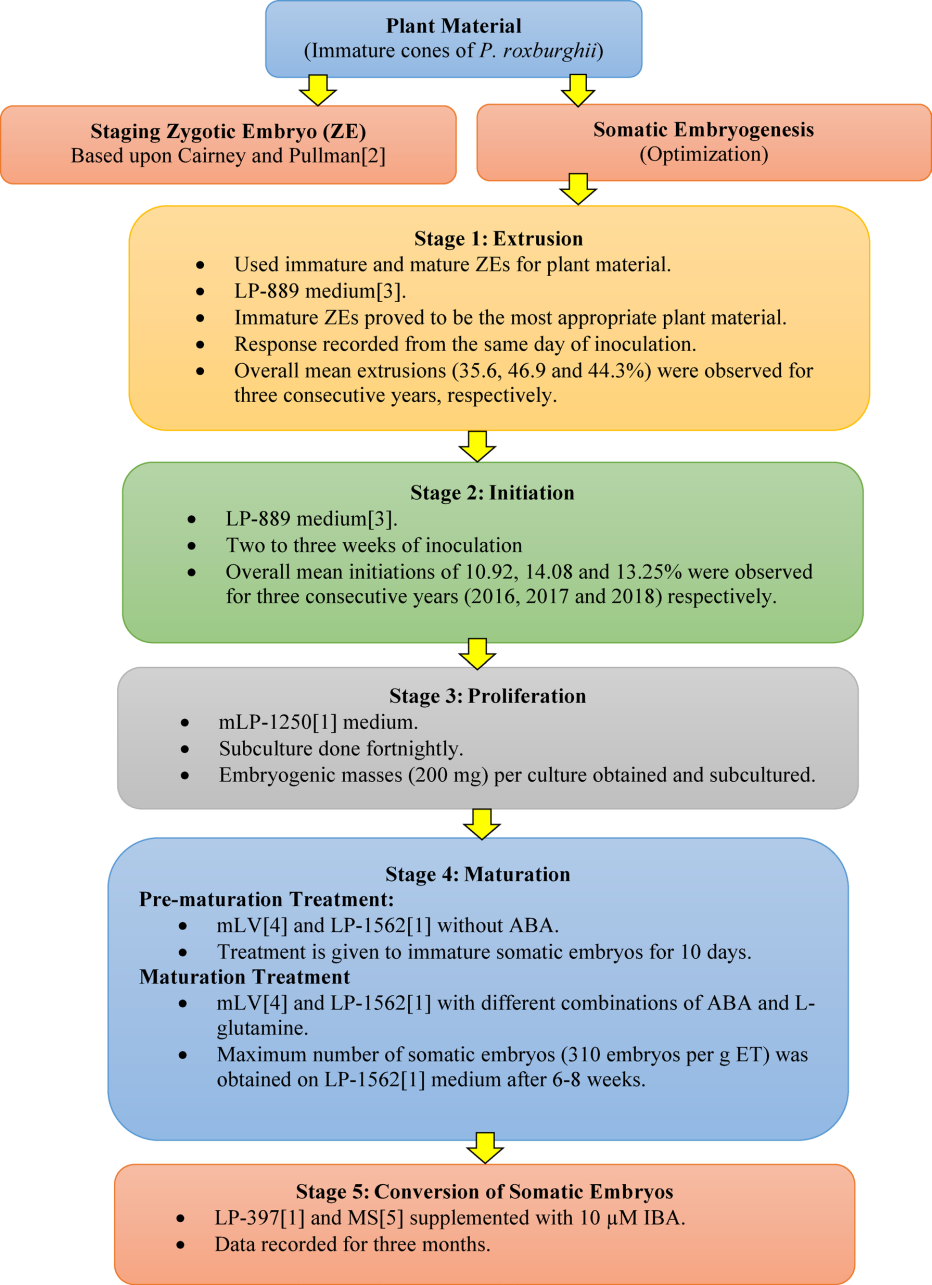
Schematic flow chart showing different steps of somatic embryogenesis of *P. roxburghii*.

In conclusion, from this initial study based on modified SE protocols originally used for *P. taeda*^3^, we suggest that somatic embryogenesis in *P. roxburghii* has a strong potential to be further developed for large-scale effective plant production. The results from this study (Fig. 11) are encouraging since somatic embryos were not only initiated at a comparatively higher rate for pines but also continued proliferation and matured within 6–8 weeks. Since the efficiency of the medium varies from species to species and the fact that even slight changes in various other factors (both environmental as well as chemical) do affect such growth phenomena considerably, it is assumed that further work with some modifications in experimental parameters may lead us to a fully workable and reproducible SE protocol suitable for large-scale plant production. Further work is hence continuing not only to account for these factors or limitations but also to open further possibilities to propagate other economically important pine tree species of this part of the world.

## Supplementary Information

Below is the link to the electronic supplementary material.

Supplementary Material 1

## Data Availability

The datasets used and/or analysed during the current study available from the corresponding author on reasonable request.
