# Supplementary material for: An improved and simplified somatic embryogenesis protocol in Chir pine (Pinus roxburghii)
Source: Sci Rep. 2025 Oct 28;15:37560. doi: 10.1038/s41598-025-20364-4 (PMC12569014; doi:10.1038/s41598-025-20364-4)
Supplement: Supplementary file 1 — Supplementary Material 1 [file 41598_2025_20364_MOESM1_ESM.docx]

Supplementary Material

Statistical Analysis

| **Table 1. Levene's test of equality of variance between fixed factors and dependent variables in *P. roxburghii*** | | | | | | | | | |
| --- | --- | --- | --- | --- | --- | --- | --- | --- | --- |
| **Extrusion** | | ***F* value** | ***df^1^*** | | | ***df^2^*** | | **Sig. <0.05** | |
|  |  | .945 | 71 | | | 144 | | 0.599 | |
| **Initiation** | | 2.784 | 71 | | | 144 | | 0.000 | |
| Tests the null hypothesis that the error variance of the dependent variable is equal across groups. | | | | | | | | | |
| **Table 2. Two-way ANOVA (multivariate analysis) of interactive effects on extrusion and initiation of somatic embryos of *P. roxburghii*** | | | | | | | | |  |
| **Source** | **Dependent Variable** | | | ***df*** | ***F* value** | | **Sig.**  **<0.05** | |  |
| Corrected Model | Extrusion | | | 71 | 586.579 | | 0.000 | |  |
|  | Initiation | | | 71 | 579.508 | | 0.000 | |  |
| Intercept | Extrusion | | | 1 | 145256.266 | | 0.000 | |  |
|  | Initiation | | | 1 | 47857.969 | | 0.000 | |  |
| Tree | Extrusion | | | 2 | 16.724 | | 0.000 | |  |
|  | Initiation | | | 2 | 3.331 | | 0.039 | |  |
| Year | Extrusion | | | 2 | 963.411 | | 0.000 | |  |
|  | Initiation | | | 2 | 256.141 | | 0.000 | |  |
| Collection date | Extrusion | | | 7 | 5379.940 | | 0.000 | |  |
|  | Initiation | | | 7 | 5448.462 | | 0.000 | |  |
| Tree × Year | Extrusion | | | 4 | 9.229 | | 0.000 | |  |
|  | Initiation | | | 4 | 20.337 | | 0.000 | |  |
| Tree × collection date | Extrusion | | | 14 | 16.422 | | 0.000 | |  |
|  | Initiation | | | 14 | 13.203 | | 0.000 | |  |
| Year × collection date | Extrusion | | | 14 | 97.544 | | 0.000 | |  |
|  | Initiation | | | 14 | 99.759 | | 0.000 | |  |
| Tree × Year × collection date | Extrusion | | | 28 | 14.100 | | 0.000 | |  |
|  | Initiation | | | 28 | 29.431 | | 0.000 | |  |
| a. R Squared = 0.997; b. R Squared = 0.997 | | | | | | | | |  |

Media

**ANNEXURE-1**

| **FORMULATION OF LP 889 MEDIUM (Pullman et al. 2003) FOR THE PREPARATION OF STOCK SOLUTIONS** | | |
| --- | --- | --- |
| **Components** | **Stock Concentration** | **Final Concentration in LP medium** |
| **Macronutrients** | **mg l^-1^ (10x)** | **mg l^-1^** |
| NH_4_NO_3_ | 200 × 10 = 2,000 | 200 |
| KNO_3_ | 9099 | 909.9 |
| KH_2_PO_4_ | 1361 | 136.1 |
| Ca (NO_3_).4H_2_O | 3000 | 300 |
| MgSO_4_.7H_2_O | 3000 | 300 |
| MgCl_2_.6H_2_O | 1017 | 101.7 |
| **Micronutrients** | **mg l^-1^ (50x)** | **mg l^-1^** |
| KI | 4.15 × 50 = 207.5 | 4.15 |
| H_3_BO_3_ | 775 | 15.5 |
| MnSO_4_.H_2_O | 525 | 10.5 |
| ZnSO_4_.7H_2_O | 733.4 | 14.668 |
| Na_2_MoO.2H_2_O | 6.25 | 0.125 |
| CuSO_4_.5H_2_O | 8.625 | 0.1725 |
| CoCl_2_.6H_2_O | 6.25 | 0.125 |
| **Iron-EDTA** | **mg l^-1^ (100x)** | **mg l^-1^** |
| FeSO_4_.7H_2_O | 13.9 × 100 = 1390 | 13.9 |
| Na_2_EDTA | 1865 | 18.65 |
| **Vitamins** | **mg l^-1^ (50x)** | **mg l^-1^** |
| Thiamine HCl | 1.0 × 50 = 50 | 1.0 |
| Pyridoxine HCl | 25 | 0.5 |
| Nicotinic acid | 25 | 0.5 |
| Glycine | 100 | 2.0 |
| **Growth Regulator** | **mg l^-1^** | |
| NAA | 2.0 (10.74 µM) | |
| BA | 0.55 (2.44 µM) | |
| Kinetin | 0.53 (2.46 µM) | |
| ABA^a^ | 1.0 (3.78 µM) | |
| **Others** | **mg l^-1^** | |
| AgNO_3_ | 3.398 | |
| _L_-Glutamine^b^ | 450 | |
| Maltose | 15,000 | |
| Myo-inositol | 20,000 | |
| Casamino acids | 500 | |
| AC | 50 | |
| Gelrite | 2,000 | |
| pH | 5.7 | |

^a^ Filter-sterilized stock solution was added to the autoclaved medium after cooling to 55-60^0^C

**NOTE:** Some adjustments were brought in LP 889. MgCl_2_.6H_2_O was omitted by adjusting the quantities of Ca (NO_3_).4H_2_O and MgSO_4_.7H_2_O to 300 mg/l each from 236.2 mg/l and 246.5 mg/l respectively. We neglected _L-_Asparagine and 8-Br-cGMP in our modified medium.

**ANNEXURE-2**

| **FORMULATION OF LP (LOBLOLLY PINE) 1250 MEDIUM FOR THE PREPARATIONOF STOCK SOLUTIONS** | | |
| --- | --- | --- |
| **Components** | **Stock Concentration** | **Final Concentration in LP medium** |
| **Macronutrients** | **mg l^-1^ (10x)** | **mg l^-1^** |
| NH_4_NO_3_ | 603.8 × 10 = 2,000 | 603.8 |
| KNO_3_ | 9099 | 909.9 |
| KH_2_PO_4_ | 1361 | 136.1 |
| Ca (NO_3_).4H_2_O | 3000 | 300 |
| MgSO_4_.7H_2_O | 3000 | 300 |
| MgCl_2_.6H_2_O | 1017 | 101.7 |
| **Micronutrients** | **mg l^-1^ (50x)** | **mg l^-1^** |
| KI | 4.15 × 50 = 207.5 | 4.15 |
| H_3_BO_3_ | 775 | 15.5 |
| MnSO_4_.H_2_O | 525 | 10.5 |
| ZnSO_4_.7H_2_O | 720 | 14.4 |
| Na_2_MoO_4_.2H_2_O | 6.25 | 0.125 |
| CuSO_4_.5H_2_O | 6.25 | 0.125 |
| CoCl_2_.6H_2_O | 6.25 | 0.125 |
| **Iron-EDTA** | **mg l^-1^ (100x)** | **mg l^-1^** |
| FeSO_4_.7H_2_O | 6.95 × 100 = 695 | 6.95 |
| Na_2_EDTA | 933 | 9.33 |
| **Vitamins** | **mg l^-1^ (50x)** | **mg l^-1^** |
| Thiamine HCl | 1.0 × 50 = 50 | 1.0 |
| Pyridoxine HCl | 25 | 0.5 |
| Nicotinic acid | 25 | 0.5 |
| Glycine | 100 | 2.0 |
| **Growth Regulator** | **mg l^-1^** | |
| 2,4-D | 1.1 (4.976 µM) | |
| BAP | 0.45 (1.995 µM) | |
| Kinetin | 0.43 (1.998 µM) | |
| ABA^a^ | 1.3 (4.91µM) | |
| **Others** | **mg l^-1^** | |
| _L_-Glutamine^b^ | 450 | |
| Sucrose | 30,000 | |
| Myo-inositol | 10,000 | |
| Casamino acids | 500 | |
| Gelrite | 2,500 | |
| pH | 5.7 | |

^a^ Filter-sterilized stock solution was added to the autoclaved medium after cooling to 55-60^0^C

**NOTE:** Some adjustments were brought in LP 1250. MgCl_2_.6H_2_O was omitted by adjusting the quantities of Ca (NO_3_).4H_2_O and MgSO_4_.7H_2_O to 300 mg/l each from 236.2 mg/l and 246.5 mg/l respectively.

**ANNEXURE-3 A**

| **FORMULATION OF LP 1562 MEDIUM (LOBLOLLY PINE) FOR THE PREPARATIONOF STOCK SOLUTIONS** | | |
| --- | --- | --- |
| **Components** | **Stock Concentration** | **Final Concentration in LP medium** |
| **Macronutrients** | **mg l^-1^ (10x)** | **mg l^-1^** |
| NH_4_NO_3_ | 200 × 10 = 2,000 | 200 |
| KNO_3_ | 4549 | 454.9 |
| KH_2_PO_4_ | 1361 | 136.1 |
| Ca (NO_3_).4H_2_O | 591 | 59.1 |
| MgSO_4_.7H_2_O | 2465 | 246.5 |
| Mg (NO_3_)_2_.6H_2_O | 2565 | 256.5 |
| MgCl_2_.6H_2_O | 1017 | 101.7 |
| **Micronutrients** | **mg l^-1^ (50x)** | **mg l^-1^** |
| KI | 4.15 × 50 = 207.5 | 4.15 |
| H_3_BO_3_ | 387.5 | 7.75 |
| MnSO_4_.H_2_O | 525 | 10.5 |
| ZnSO_4_.7H_2_O | 720 | 14.4 |
| Na_2_MoO_4_.2H_2_O | 6.25 | 0.125 |
| CuSO_4_.5H_2_O | 6.25 | 0.125 |
| CoCl_2_.6H_2_O | 6.25 | 0.125 |
| **Iron-EDTA** | **mg l^-1^ (100x)** | **mg l^-1^** |
| FeSO_4_.7H_2_O | 41.7 × 100 = 4170 | 41.7 |
| Na_2_EDTA | 5595 | 55.95 |
| **Vitamins** | **mg l^-1^ (50x)** | **mg l^-1^** |
| Thiamine HCl | 1.0 × 50 = 50 | 1.0 |
| Pyridoxine HCl | 25 | 0.5 |
| Nicotinic acid | 25 | 0.5 |
| Glycine | 100 | 2.0 |
| **Growth Regulator** | **mg l^-1^** | |
| ABA^a^ | 5.2 (21.11µM) | |
| **Others** | **mg l^-1^** | |
| L-Glutamine^b^ | 450 | |
| Maltose | 20,000 | |
| Myo-inositol | 100 | |
| Casamino acids | 500 | |
| Gelrite | 2,500 | |
| pH | 5.7 | |

^a,b^ Filter-sterilized stock solution was added to the autoclaved medium after cooling to 55-60^0^C

**ANNEXURE-3 C**

| **FORMULATION OF mLV (Litvay et al. 1985) MEDIUM FOR THE PREPARATIONOF STOCK SOLUTIONS** | | |
| --- | --- | --- |
| **Components** | **Stock Concentration** | **Final Concentration in mLV medium** |
| **Macronutrients** | **mg l^-1^ (10x)** | **mg l^-1^** |
| NH_4_ NO_3_ | 825 × 10 = 8250 | 825 |
| KNO_3_ | 9500 | 950 |
| KH_2_PO_4_ | 1700 | 170 |
| CaCl_2_.2H_2_O | 830 | 8.3 |
| MgSO_4_.7H_2_O | 4516 | 451.69 |
| **Micronutrients** | **mg l^-1^ (50x)** | **mg l^-1^** |
| KI | 4.15 × 50 = 207.5 | 4.15 |
| H_3_BO_3_ | 1550 | 31 |
| MnSO_4_.4H_2_O | 1050 | 21 |
| ZnSO_4_.7H_2_O | 2150 | 43 |
| Na_2_MoO_4_.2H_2_O | 62.5 | 1.25 |
| CuSO_4_.5H_2_O | 25 | 0.5 |
| CoCl_2_.6H_2_O | 6.25 | 0.125 |
| **Iron-EDTA** | **mg l^-1^ (100x)** | **mg l^-1^** |
| FeSO_4_.7H_2_O | 27.8 × 100 = 2780 | 27.8 |
| Na_2_EDTA | 3730 | 37.3 |
| **Vitamins** | **mg l^-1^ (50x)** | **mg l^-1^** |
| Thiamine HCl | 0.1 × 50 = 5 | 0.1 |
| Pyridoxine HCl | 5 | 0.1 |
| Nicotinic acid | 25 | 0.5 |
| **Growth Regulators** | **µM** | |
| ABA | 80 | |
| **Others** | **mg l^-1^** | |
| _L_-Glutamine^a^ | 0.625 | |
| Myo-inositol | 100 | |
| Agar | 9,000 | |
| pH | 5.7-5.8 | |

^a^ Filter-sterilized stock solution was added to the autoclaved medium after cooling to 55-60^0^C

**ANNEXURE-4 A**

| **FORMULATION OF LP (LOBLOLLY PINE) 397 MEDIUM FOR THE PREPARATIONOF STOCK SOLUTIONS** | | |
| --- | --- | --- |
| **Components** | **Stock Concentration** | **Final Concentration in LP medium** |
| **Macronutrients** | **mg l^-1^ (10x)** | **mg l^-1^** |
| NH_4_NO_3_ | 206.3× 10 = 2,063 | 206.3 |
| KNO_3_ | 4549 | 1170 |
| KH_2_PO_4_ | 1361 | 85 |
| CaCl_2_.2H_2_O | 2200 | 220 |
| MgSO_4_.7H_2_O | 1855 | 185.5 |
| **Micronutrients** | **mg l^-1^ (50x)** | **mg l^-1^** |
| KI | 0.415 × 50 = 20.75 | 0.415 |
| H_3_BO_3_ | 155 | 3.1 |
| MnSO_4_.H_2_O | 422.5 | 8.45 |
| ZnSO_4_.7H_2_O | 215 | 4.3 |
| Na_2_MoO_4_.2H_2_O | 6.25 | 0.125 |
| CuSO_4_.5H_2_O | 12.5 | 0.25 |
| CoCl_2_.6H_2_O | 6.25 | 0.125 |
| **Iron-EDTA** | **mg l^-1^ (100x)** | **mg l^-1^** |
| FeSO_4_.7H_2_O | 13.93 × 100 = 1393 | 13.93 |
| Na_2_EDTA | 1865 | 18.65 |
| **Vitamins** | **mg l^-1^ (50x)** | **mg l^-1^** |
| Thiamine HCl | 1.0 × 50 = 50 | 1.0 |
| Pyridoxine HCl | 25 | 0.5 |
| Nicotinic acid | 25 | 0.5 |
| Glycine | 100 | 2.0 |
| **Growth Regulator** | **mg l^-1^** | |
| IBA | 2.03 (10 µM) | |
| **Others** | **mg l^-1^** | |
| Sucrose | 20,000 | |
| Myo-inositol | 100 | |
| Activated carbon | 2500 | |
| Agar | 8,000 | |
| pH | 5.7 | |

**ANNEXURE-4 B**

| **FORMULATION OF MS (MURASHIGE AND SKOOG, 1962) MEDIUM FOR THE PREPARATIONOF STOCK SOLUTIONS** | | |
| --- | --- | --- |
| **Components** | **Stock Concentration** | **Final Concentration in MS medium** |
| **Macronutrients** | **mg l^-1^ (10x)** | **mg l^-1^** |
| NH_4_NO_3_ | 10 × 1650 = 16500 | 1650 |
| KNO_3_ | 19000 | 1900 |
| MgSO_4_.7H_2_O | 3700 | 370 |
| CaCl_2_.2H_2_O | 4400 | 440 |
| KH_2_PO_4_ | 1700 | 170 |
| **Micronutrients** | **mg l^-1^ (50x)** | **mg l^-1^** |
| MnSO_4_.H_2_O | 50 × 22.3 = 1115 | 22.3 |
| ZnSO_4_.7H_2_O | 430 | 8.6 |
| H_3_BO_3_ | 310 | 6.2 |
| KI | 41.5 | 0.83 |
| Na_2_MoO_4_.2H_2_O | 12.5 | 0.25 |
| CuSO_4_.5H_2_O | 1.25 | 0.025 |
| CoCl_2_.6H_2_O | 1.25 | 0.025 |
| **Iron-EDTA** | **mg l^-1^ (100x)** | **mg l^-1^** |
| Na_2_EDTA | 36.2 × 100 = 3620 | 36.2 |
| FeSO_4_.7H_2_O | 2780 | 27.8 |
| **Vitamins** | **mg l^-1^ (50x)** | **mg l^-1^** |
| Glycine | 2.0 × 50 = 100 | 2.0 |
| Nicotinic acid | 25 | 0.5 |
| Pyridoxine HCl | 25 | 0.5 |
| Thiamine HCl | 5 | 0.1 |
| **Others** | **mg l^-1^** | |
| Sucrose | 30000 | |
| Myo-inositol | 100 | |
| Agar | 8000 | |
| pH | 5.8 | |

**Related Figures**

**Fig. 1** Average extrusion and initiation frequencies of *P. roxburghii* by using immature ZEs. Each peak represents the mean (%) response of the ZEs for extrusion and initiation during three years of experiments. Values (±SE) are means of 240 replications per year (5-6 seeds per replicate)


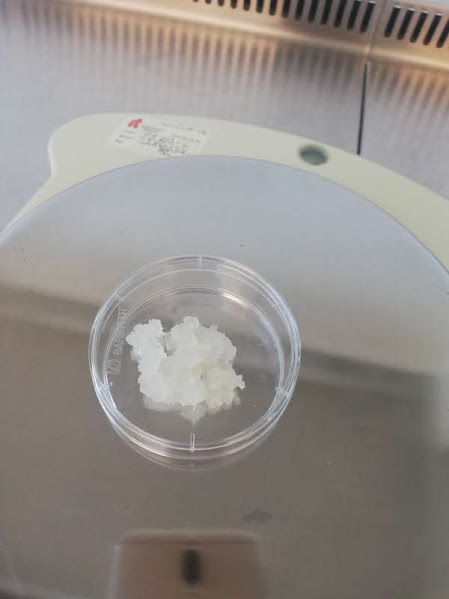


**Fig. 2** Embryonic tissue to be cultured on medium for fresh mass increase measurement


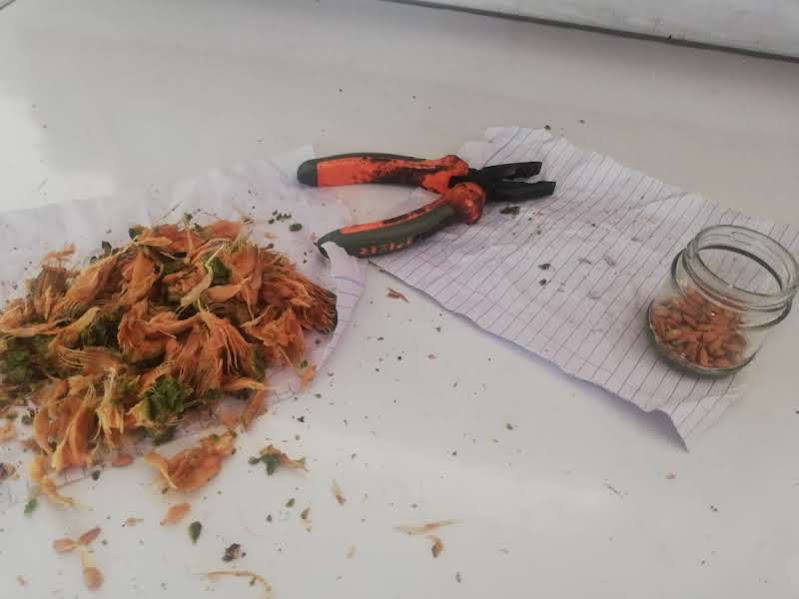

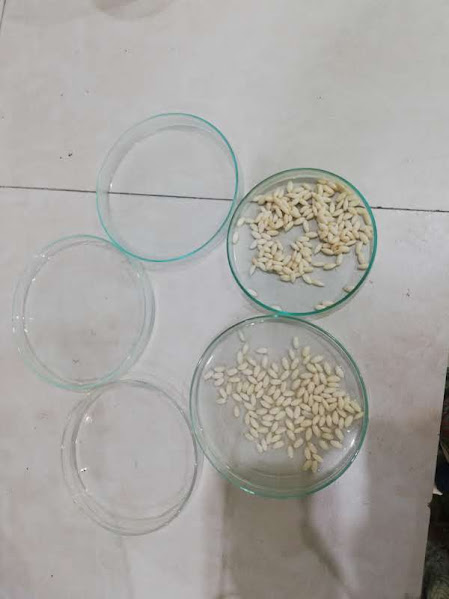


**Fig. 3 (a-b)** Extraction of seeds from *P. roxburghii* cones (a). Isolation of megagametophytes to study developing zygotic embryo stage. ). (Scale Bar a = 4.5 cm, b =5 cm)*.*


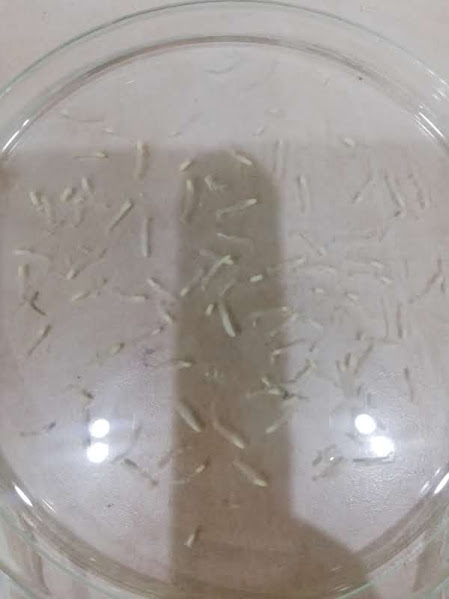

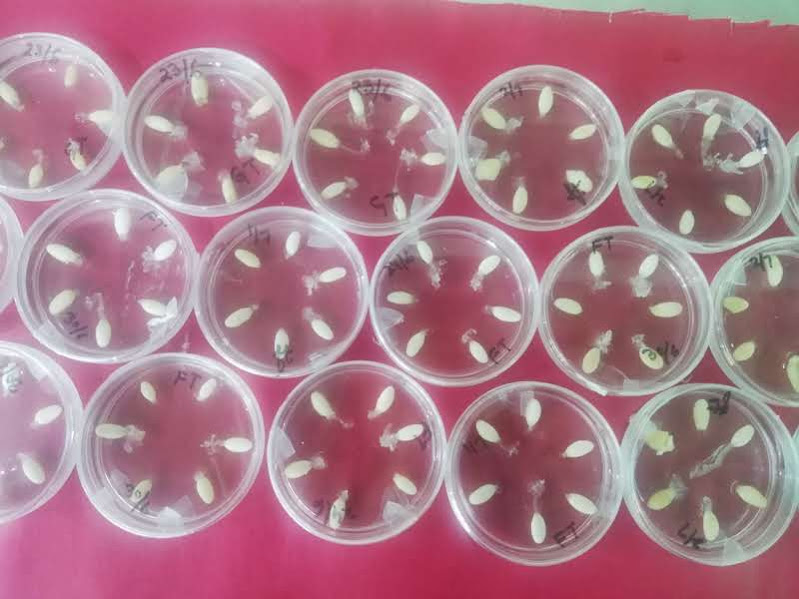


**Fig. 4 (a-b)** Isolated zygotic embryos from excised megagametophytes (a). Initiated embryonic tissue from micropylar end of megagametophytes (Scale Bar a = 2.5 cm, b =3 cm)*.*


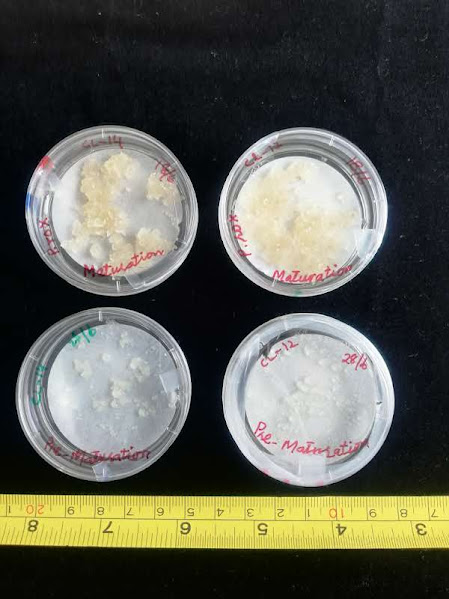

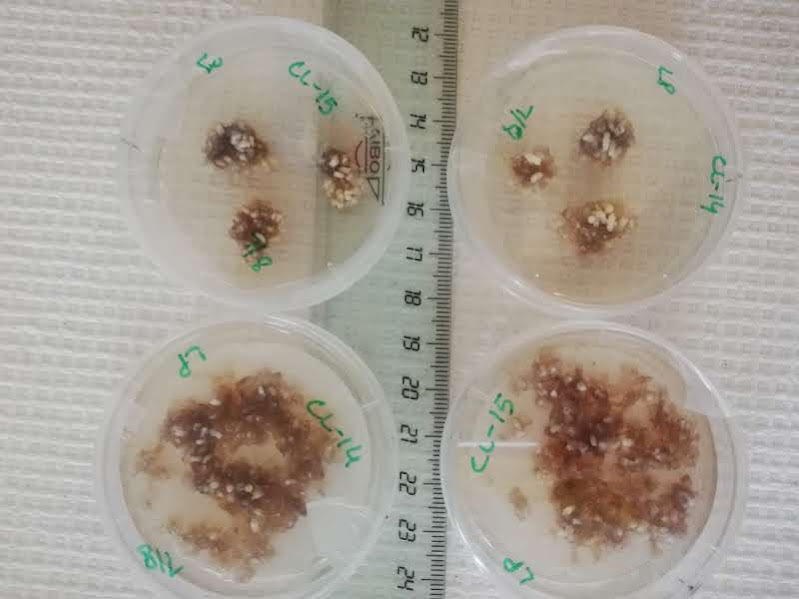


**Fig. 5(a-b)** Embryonic tissue on pre-maturation and maturation medium on filter paper discs at the start of experiment (a). Embryonic tissue solid clumps (upper plates) and suspension (lower plates) on filter paper discs forming somatic embryos

**Stage 4: Maturation**

**Pre maturation Treatment:**

- mLV (Litvay et al*.* 1985) and mLP-1562 (Pullman and Bucalo 2011) without ABA
- Treatment given to immature somatic embryos for 10 days

**Maturation Treatment**

- mLV (Litvay et al*.* 1985) and mLP-1562 (Pullman and Bucalo 2011) with different combinations of ABA and L-glutamine
- Maximum number of somatic embryos (310 embryos per g ET) was obtained on mLP-1562 medium after 6-8 weeks

**Stage 5: Germination**

- LP-397 (Pullman and Bucalo 2011) and MS (Murashige and Skoog 1962) supplemented with 10 µM IBA
- Data recorded for three months

**Stage 3: Proliferation**

- mLP-1250 medium (Pullman and Bucalo 2011)
- Subculture done fortnightly
- 200 mg embryogenic masses per culture obtained and subcultured

**Stage 2: Initiation**

- LP-889 medium (Pullman et al. 2003)
- 2-3 weeks of inoculation
- overall mean initiations 10.92%, 14.08% and 13.25% were observed for three consecutive years respectively

**Stage 1: Extrusion**

- Used immature and mature ZEs for plant material
- LP-889 medium (Pullman et al. 2003)
- Immature ZEs proved to be the most appropriate plant material
- Response recorded from the same day of inoculation
- Overall mean extrusions 35.6%, 46.9% and 44.3% were observed for three consecutive years respectively

**Somatic Embryogenesis**

(Optimization)

**Staging Zygotic Embryo (ZE)**

Idea based upon Cairney and Pullman (2007)

**Plant Material**

(Immature cones of *P. roxburghii*)
